# Trends in the management and prognosis of mucinous borderline ovarian tumors: analysis of 12,766 cases from the JSOG Gynecologic Tumor Registry (2004–2018)

**DOI:** 10.1007/s10147-026-03008-z

**Published:** 2026-03-19

**Authors:** Hideki Tokunaga, Yusuke Shibuya, Wataru Yamagami, Fumiaki Takahashi, Eiko Yamamoto, Yoshihito Yokoyama, Kiyoshi Yoshino, Kei Kawana, Satoru Nagase

**Affiliations:** 1 Division of Obstetrics and Gynecology, Faculty of Medicine, Tohoku Medical and Pharmaceutical University, Miyagi, Japan; 2 Department of Gynecology, Tohoku University Hospital, Miyagi, Japan; 3 Department of Obstetrics and Gynecology, Keio University School of Medicine, Tokyo, Japan; 4 Department of Information Science, Iwate Medical University, Iwate, Japan; 5 Department of Healthcare Administration, Nagoya University Graduate School of Medicine, Nagoya, Japan; 6 Department of Obstetrics and Gynecology, Hirosaki University Graduate School of Medicine, Aomori, Japan; 7 Department of Obstetrics and Gynecology, University of Occupational and Environmental Health, Fukuoka, Japan; 8 Department of Obstetrics and Gynecology, Nihon University School of Medicine, Tokyo, Japan; 9 Department of Obstetrics and Gynecology, Yamagata University Faculty of Medicine, Yamagata, Japan

**Keywords:** Mucinous borderline ovarian tumor, JSOG registry, Omentectomy, Survival analysis, Fertility-sparing surgery, Nationwide cohort

## Abstract

**Background:**

Mucinous borderline ovarian tumors (MBOTs) are rare neoplasms with excellent prognosis, yet the optimal surgical extent remains controversial. No large-scale study in Japan has evaluated treatment trends and prognostic factors for MBOTs. This study aimed to clarify their clinicopathological features, management patterns, and survival outcomes using a nationwide registry.

**Methods:**

Data were obtained from the Japan Society of Obstetrics and Gynecology Gynecologic Tumor Registry, including 96,476 ovarian tumors treated between 2004 and 2018. Among them, 12,766 MBOT cases were identified. Surgical procedures—hysterectomy, omentectomy, lymphadenectomy, and adjuvant chemotherapy—were analyzed. Survival analyses of 8564 cases with complete prognostic data were performed using Kaplan–Meier and Cox proportional hazards models.

**Results:**

Over 90% of MBOTs were stage I, and the median age was 52 years. Hysterectomy was performed in 50.8%, omentectomy in 57.9% (2015–2018 subset), and lymphadenectomy in 7.6%. Only 2.6% received adjuvant chemotherapy. The 5-year overall survival exceeded 95%. Multivariate analysis identified age ≥ 50 years (HR 2.5, 95% CI 1.8–3.6) and stage IC (HR 2.7, 95% CI 1.9–3.6) as independent adverse factors. Omentectomy showed a marginal survival benefit (HR 0.6, *p* = 0.05), whereas hysterectomy, lymphadenectomy, and chemotherapy conferred no advantage. Chemotherapy correlated with poorer outcomes, likely due to confounding by indication.

**Conclusions:**

This nationwide cohort—the largest MBOT series reported to date—demonstrates conservative management with excellent prognosis in Japan. Radical surgery and chemotherapy provide no survival benefit, whereas fertility-sparing surgery appears appropriate for younger patients.

## Introduction

In addition to bilateral adnexal resection, total hysterectomy, and omentectomy for malignant ovarian tumors, pelvic and para-aortic lymph node dissection, ascitic (peritoneal) cytology, and intraperitoneal exploration are performed to determine the extent of disease and assess advanced stages. Some distant metastases are confirmed pathologically, but most are identified through diagnostic imaging.

For borderline ovarian tumors, routine lymph node dissection is not recommended [1]. Apart from the LION study [2], no randomized controlled trials have evaluated the necessity of lymphadenectomy in advanced ovarian cancer. Current recommendations are, therefore, based on the retrospective detection rates of occult metastases, meta-analyses, and their impact on prognosis [1].

In summary, it is only weakly recommended to perform basic surgical staging procedures beyond resection of the affected adnexa. To date, no nationwide or long-term large-scale study has evaluated mucinous borderline ovarian tumors in Japan, and real-world treatment strategies remain largely undefined.

The Japan Society of Obstetrics and Gynecology (JSOG) began registering cases of borderline and malignant ovarian tumors in the Gynecologic Tumor Registry (GTR) starting in 1998. This registry collects data on clinicopathological features and survival outcomes, with follow-up surveys performed three and five years after registration. Histological classification follows the WHO system, and staging is based on the International Federation of Gynecology and Obstetrics (FIGO) classification; both are updated in accordance with each revision.

In the present study, we analyzed large-scale data from 2004 to 2018, when five-year prognostic follow-up was completed, to investigate treatment trends in mucinous borderline ovarian tumors and to evaluate the prognostic impact of surgical procedures other than adnexal resection.

## Materials and methods

### Patients

This study included 96,476 patients with ovarian tumors treated at medical facilities across Japan and registered in the Japan Society of Obstetrics and Gynecology (JSOG) Gynecologic Tumor Registry (GTR) between 2004 and 2018. Major hospitals throughout Japan participate in this registry, which is estimated to cover approximately 70–80% of all ovarian cancer cases nationwide.

After receiving approval from the Ethics Committees of the JSOG (approval no. 17) and Keio University School of Medicine (approval no. 20170261), data on clinicopathological characteristics and survival outcomes were collected. Patients who did not undergo surgery or who received preoperative chemotherapy were excluded from the prognostic analysis. Cases with incomplete clinical information—such as missing stage data or unavailable prognostic outcomes—were also excluded.

### Methods

**Fig. 1:**
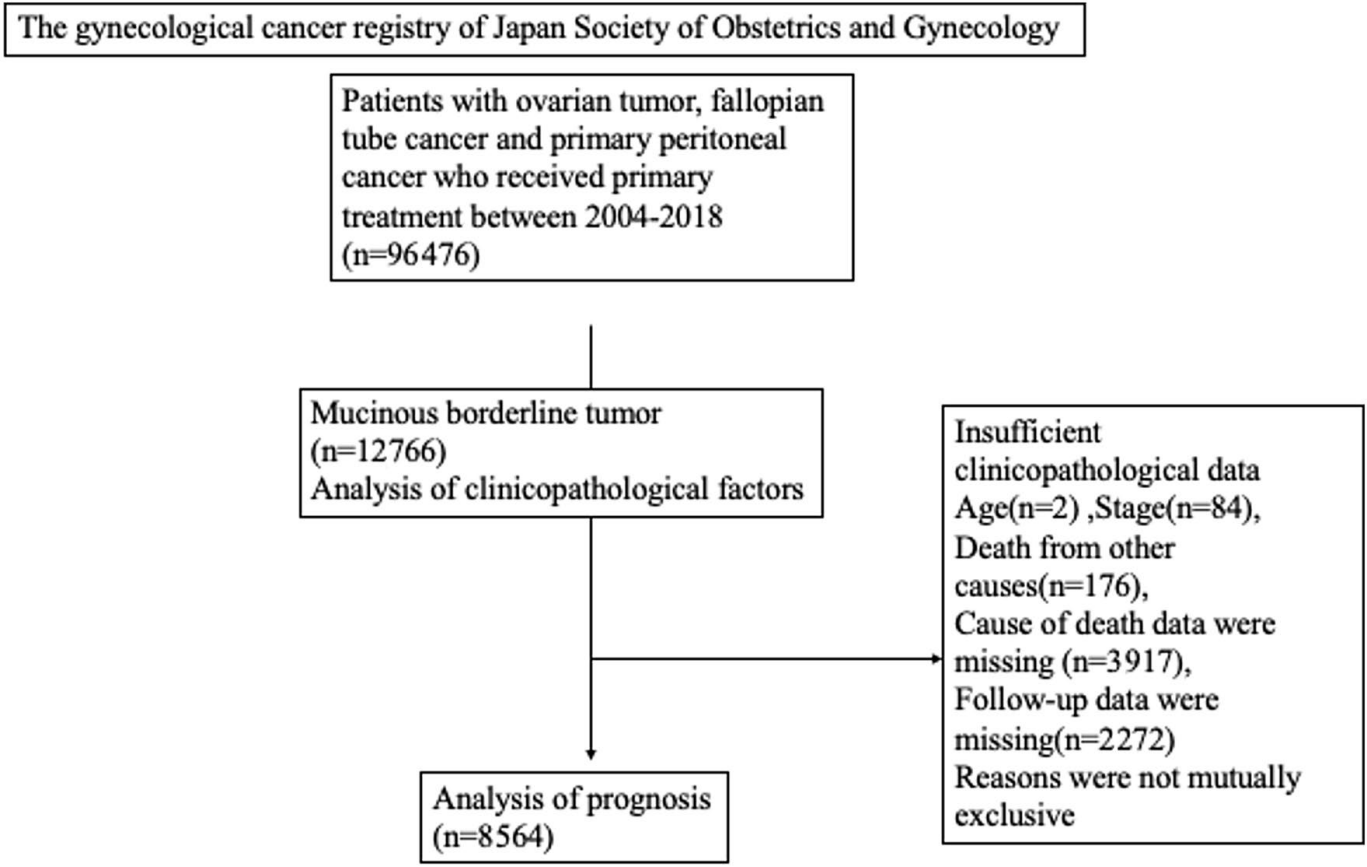
Flow diagram of this study. Patients with ovarian, fallopian tube, and primary peritoneal tumors registered in the JSOG gynecologic cancer registry between 2004 and 2018 (*n* = 96,476) were screened. After exclusion of cases with insufficient clinicopathologic or follow-up data, 12,766 mucinous borderline ovarian tumors (MBOTs) were included in the clinicopathologic analysis, and 8564 cases were eligible for prognostic evaluation

The study flow diagram illustrates the selection and stratification of the cohort (Fig. 1). A subset of 8564 cases with complete prognostic information was included in the survival analysis.

The registry data included: age at treatment initiation, FIGO stage (1988 or 2014), pTNM classification according to the FIGO system, whether preoperative chemotherapy was administered, surgical procedures (adnexectomy, hysterectomy, omentectomy, and lymphadenectomy), residual tumor status (surgical completeness), sites of distant metastasis, whether adjuvant chemotherapy or second-look surgery was performed, and ypTNM classification. Follow-up surveys are conducted three and five years after the year of registration to determine disease-free survival, alive-with-disease, and death outcomes. Since 2017, the registry has been updated to reflect the WHO 2014 histological classification, which redefined mucinous tumors as intestinal type and seromucinous type. Data prior to 2016 followed the WHO 2003 classification, which did not distinguish between intestinal and endocervical types; therefore, these earlier data cannot be directly reclassified under the WHO 2014 system. Beginning in 2015, staging was based on FIGO 2014; however, before 2014, the size of lymph node metastases was not recorded, preventing precise stage conversion. Until 2014, surgical procedures were categorized only as “adnexectomy” or “radical surgery,” and information on omentectomy was unavailable. Because “radical surgery” was defined as including hysterectomy, the presence or absence of hysterectomy could still be determined. For this study, seromucinous tumors diagnosed after 2015 were excluded from the survival analysis.

### Statistical analysis

All statistical analyses were performed using JMP software, version 19.0.1 (SAS Institute Inc., Cary, NC, USA). The univariate analyses for overall survival (OS) were conducted using the Kaplan–Meier method and the log-rank test. The multivariate analyses were performed using the Cox proportional hazards model, incorporating available prognostic factors (age, surgical stage, surgical procedure, and adjuvant chemotherapy). To minimize selection bias, additional analyses were conducted using multivariate logistic regression and Cox regression with propensity score matching. In all analyses, a *p* value < 0.05 was considered statistically significant.

## Results

**Fig. 2:**
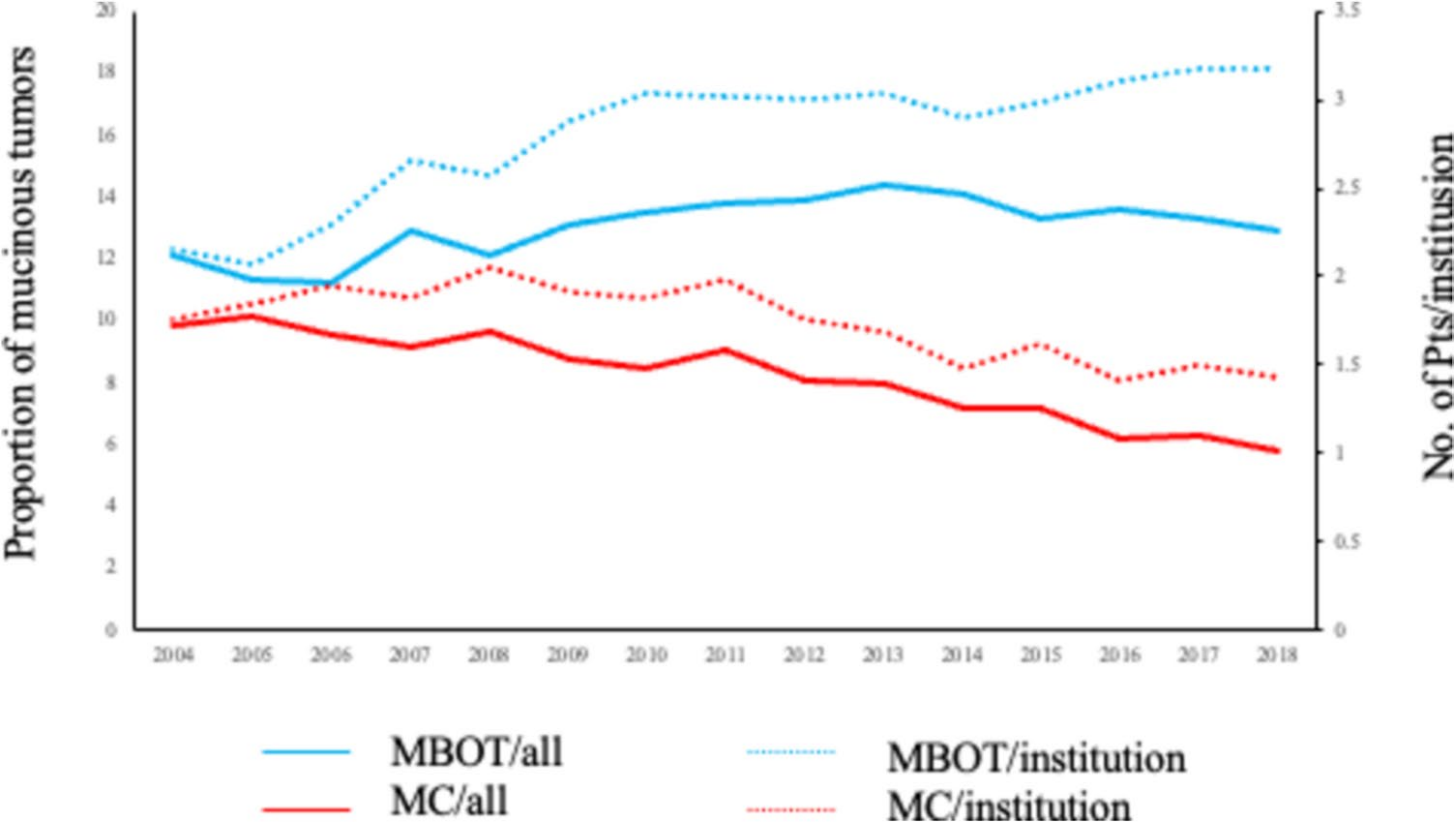
Annual trends of mucinous tumors among ovarian neoplasms. MBOT/all and MC/all indicate the proportions of mucinous borderline tumors and mucinous carcinomas among all registered ovarian tumors, respectively. MBOT/institution and MC/institution indicate the number of patients per participating institution

Between 2004 and 2018, a total of 96,476 ovarian neoplasms were registered from 216 to 398 institutions (Table 1). Mucinous borderline ovarian tumors (MBOTs) accounted for 12,766 cases (13.2%), showing a steady increase in absolute number—from 466 cases in 2004 to 1192 in 2018. Seromucinous borderline tumors were reported in 296 patients in 2017 and 305 in 2018. Both the number of participating institutions and registered cases increased over time, indicating broader registry coverage and improved data quality. The proportion of MBOTs rose slightly from 12.1% in 2004 to a peak of 14.4% in 2013, then stabilized around 13% thereafter. The average number of MBOT cases per institution increased from 2.16 to 3.17 per year, suggesting both improved detection and wider participation (Fig. 2). By contrast, the number of mucinous carcinomas (MCs) ranged from 350 to 620 cases annually, without a clear upward trend. The proportion of MCs among all ovarian tumors declined from approximately 10% (2004–2006) to 6% (2017–2018), and the number of MCs per institution decreased from 1.9 to 1.4 per year, suggesting a relative reduction in the malignant mucinous component among newly registered ovarian tumors.

**Table 1:** Annual trend in the number and proportion of mucinous borderline ovarian tumors among all ovarian tumors (2004–2018)

Year	2004	2005	2006	2007	2008	2009	2010	2011	2012	2013	2014	2015	2016	2017	2018	Total
Registered patients	3853	3490	4041	4359	4820	5277	5678	6102	6902	7718	7860	8646	9090	9383	9257	96,476
Mucinous borderline tumor	466	397	452	562	585	694	769	840	958	1114	1107	1149	1236	1245	1192	12,766
Seromucinous borderline tumor														296	305	
Mucinous carcinoma	379	355	385	398	466	461	478	551	559	619	568	622	565	586	540	
Institutions	216	192	197	212	227	241	254	279	319	366	382	386	398	393	376

### Patient characteristics

Table 2 summarizes the clinicopathologic characteristics of patients with MBOT. The median age was 52 years (range, 11–97) between 2004 and 2014, and 53 years (range, 11–96) between 2015 and 2018. Most tumors were FIGO stage I (68.5% under 1988 criteria; 64.6% under 2014 criteria), and higher-stage disease (stage II–IV) was rare (< 5%). Hysterectomy was performed in 6484 cases (50.8%), whereas 6282 patients (49.2%) did not undergo hysterectomy. Omentectomy (data available for 2015–2018) was performed in 2791 cases (57.9%). Lymphadenectomy was carried out in 973 cases (7.6%) and adjuvant chemotherapy in 334 patients (2.6%), reflecting the indolent nature of MBOTs. Regarding outcomes, 8273 patients (64.8%) were alive without disease, 131 were alive with disease, 212 had died of disease, and 176 had died of other causes; outcomes were unknown for 3917 patients.

**Table 2:** Distribution of clinicopathologic characteristics of patients with MBOT (age, FIGO stage, and surgical procedures)

2004_2014	7944		2015_2018	4822	
Age	52 (11–97)			53 (11–96)	
FIGO stage (1988)	*n*	%	FIGO stage (2014)	*n*	%
I			I		
Ia	5439	68.5	IA	3114	64.6
Ib	91	1.1	IB	44	0.9
Ic			IC		
Ic(a)	558	7.0	IC1	988	20.5
Ic(b)	1427	18.0	IC2	398	8.3
Ic(1)	48	0.6	IC3	143	3.0
Ic(2)	121	1.5	II		
II			IIA	14	0.3
IIa	10	0.1	IIB	25	0.5
IIb	13	0.2	III		
IIc			IIIA1	1	0.0
IIc(a)	22	0.3	IIIA2	8	0.2
IIc(b)	17	0.2	IIIB	19	0.4
IIc(1)	3	0.0	IIIC	20	0.4
IIc(2)	6	0.1	IVA	5	0.1
III			IVB	2	0.0
IIIa	20	0.3	NAC	3	0.1
IIIb	27	0.3	Unknown	38	0.8
IIIc	83	1.0			
IV	16	0.2			
NAC	24	0.3			
Unknown	19	0.2			

Surgical procedure	
Hysterectomy	
Yes	6484
No	6282
Omentectomy	
2004_2014	N/A
2015_2018	
Yes	2791
No	2031
Lymph node dissection	
Yes	973
No	11,793
Adjuvant chemotherapy	
Yes	334
No	12,432
Prognosis	
Alive	8273
Alive with disease	131
Dead	212
Death from other causes	176
Unknown	3917

*NAC* neoadjuvant chemotherapy

### Surgical procedures and trends

The proportion of patients undergoing hysterectomy increased gradually until approximately 2013 and then plateaued (Table 3). Hysterectomy was predominantly performed in patients aged ≥ 50 years, who accounted for approximately 52% of all cases. Among younger women (< 40 years), fertility-sparing surgery (without hysterectomy) was more common, consistent with current clinical practice trends.

**Table 3:** Trends in hysterectomy among patients with mucinous borderline ovarian tumors

Age	10 s	20 s	30 s	40 s	50 s	60 s	70 s	80 s	90 s	N/A	Total
Yes	1	31	340	1439	1794	1616	952	298	11	2	6484
No	266	1166	1569	874	689	743	593	337	45	0	6282
Total	267	1197	1909	2313	2483	2359	1545	635	56	2	12,766

Year	2004	2005	2006	2007	2008	2009	2010	2011	2012	2013	2014	2015	2016	2017	2018	Total
Yes	203	179	200	228	248	321	369	429	522	565	616	594	648	683	679	6282
No	263	218	252	334	337	373	400	411	436	549	491	555	588	562	513	6484
Total	466	397	452	562	585	694	769	840	958	1114	1107	1149	1236	1245	1192	12,766

≥ 50 years																
Year	2004	2005	2006	2007	2008	2009	2010	2011	2012	2013	2014	2015	2016	2017	2018	Total
Yes	128	129	145	171	177	240	260	329	376	395	456	414	458	484	510	4672
No	88	77	76	113	127	144	151	165	164	203	190	214	233	246	216	2407
Total	216	206	221	284	304	384	411	494	540	598	646	628	691	730	726	7079

In the subset of 4822 patients (2015–2018), 2791 (57.9%) underwent omentectomy (Table 4). Omentectomy was more frequent in patients aged ≥ 40 years and in those with higher FIGO stages (particularly IC1–IC3). Yearly data revealed a modest increase from 52.5% (2015) to 62.4% (2018), suggesting growing adherence to comprehensive surgical staging.

**Table 4:** Omentectomy rates by age, year and FIGO stage (2015–2018 subset)

Age	10 s	20 s	30 s	40 s	50 s	60 s	70 s	80 s	90 s	Total
Yes	33	169	285	592	587	604	392	124	5	2791
No	63	235	350	320	315	330	240	156	22	2031
Total	96	404	635	912	902	934	632	280	27	4822

Year	2015	2016	2017	2018	Total
Yes	604	713	730	744	2791
No	545	523	515	448	2031
Total	1149	1236	1245	1192	4822

Stage	IA	IB	IC1	IC2	IC3	IIA	IIB	IIIA1	IIIA2	IIIB	IIIC	IVA	IVB	N/A	Total
Yes	1759	27	536	267	115	11	19	1	7	11	15	5	2	16	2791
No	1355	17	452	131	28	3	6	0	1	8	5	0	0	25	2031
Total	3114	44	988	398	143	14	25	1	8	19	20	5	2	41	4822

Lymphadenectomy was performed in 973 cases (7.6%), showing no apparent upward trend during the 15-year period (Table 5). The proportion remained below 10% even after 2010, indicating that lymphadenectomy is not routinely performed for MBOTs. Most procedures were conducted in women aged 40–60 years.

**Table 5:** Lymphadenectomy rates by age and year group

Age	10 s	20 s	30 s	40 s	50 s	60 s	70 s	80 s	90 s	N/A	Total
Yes	2	34	116	221	265	214	110	11	0	0	973
No	265	1163	1793	2092	2218	2145	1435	624	56	2	11,793
Total	267	1197	1909	2313	2483	2359	1545	635	56	2	12,766

Year	2004	2005	2006	2007	2008	2009	2010	2011	2012	2013	2014	2015	2016	2017	2018	Total
Yes	42	41	42	63	56	58	73	66	81	94	95	75	84	65	38	973
No	424	356	410	499	529	636	696	774	877	1020	1012	1074	1152	1180	1154	11,793
Total	466	397	452	562	585	694	769	840	958	1114	1107	1149	1236	1245	1192	12,766

Adjuvant chemotherapy was administered in 334 patients (2.6%) overall (Table 6), without a significant temporal increase between 2004 and 2018. Chemotherapy was mainly used for stage IC–III disease, whereas stage IA/IB patients rarely received it. Among those treated in 2015–2018, only 91 patients (1.9%) received chemotherapy, reflecting a growing trend toward surgery alone for borderline tumors.

**Table 6:** Proportion of patients receiving adjuvant chemotherapy and corresponding age, year, and FIGO stage distribution

Age	10 s	20 s	30 s	40 s	50 s	60 s	70 s	80 s	90 s	N/A	Total
Yes	5	25	36	70	95	66	30	5	1	1	334
None	262	1172	1873	2243	2388	2293	1515	630	55	1	12,432
Total	267	1197	1909	2313	2483	2359	1545	635	56	2	12,766

Year	2004	2005	2006	2007	2008	2009	2010	2011	2012	2013	2014	2015	2016	2017		
Yes	43	14	15	17	15	30	25	14	26	20	24	26	26	19	20	334
No	423	383	437	545	570	664	744	826	932	1094	1083	1123	1210	1226	1172	12,432
Total	466	397	452	562	585	694	769	840	958	1114	1107	1149	1236	1245	1192	12,766

2004_2014												
Stage	Ia	Ib	Ic	IIa	IIb	IIC	IIIa	IIIb	IIIc	IV	N/A	Total
Yes	60	5	125	2	1	13	5	5	13	6	8	243
No	5379	86	2029	8	12	35	15	22	70	10	35	7701
Total	5439	91	2154	10	13	48	20	27	83	16	43	7944
2015_2018												
Stage	IA	IB	IC	IIA	IIB	IIIA	IIIB	IIIC	IVA	IVB	N/A	
Yes	18	1	39	2	5	2	6	10	3	2	3	91
No	3096	43	1490	12	20	7	13	10	2	0	38	4731
Total	3114	44	1529	14	25	9	19	20	5	2	41	4822

### Survival analysis

**Fig. 3:**
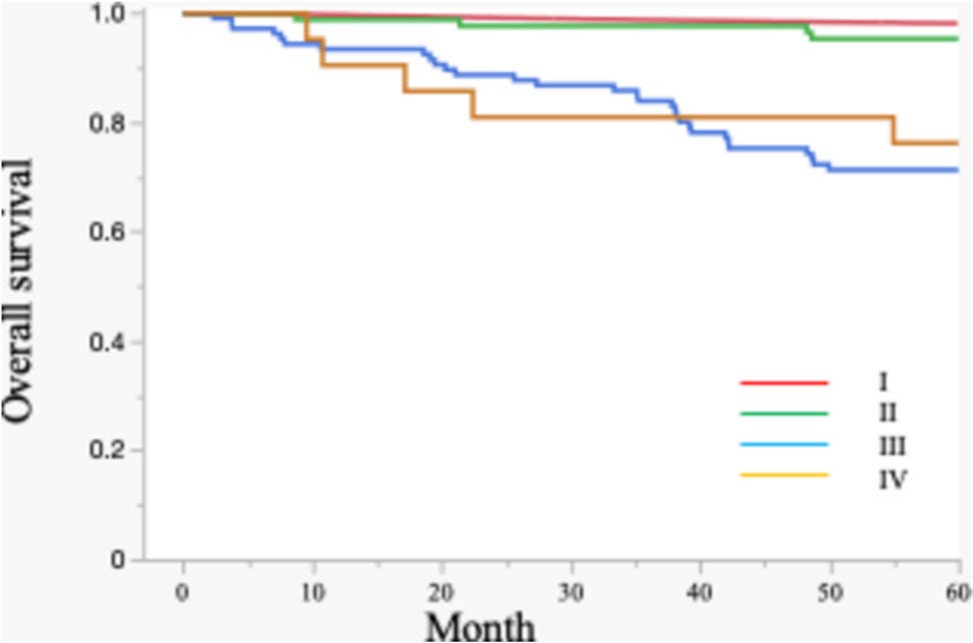
Overall survival curves of mucinous borderline ovarian tumors according to FIGO stage

The Kaplan–Meier curve (Fig. 3) demonstrated excellent long-term survival for MBOT patients, with a five-year overall survival rate exceeding 95%, consistent with the indolent behavior of these tumors.

Only a small number of deaths were observed, indicating very low disease-specific mortality.

In univariate analysis (Table 7), both older age (≥ 50 years) and higher FIGO stage (IC) were significantly associated with poorer overall survival (HR = 2.2, 95% CI 1.6–3.1, *p* < 0.0001; and HR = 2.7, 95% CI 2.0–3.7, *p* < 0.0001, respectively). Omentectomy showed a marginal trend toward improved survival (HR = 0.6, 95% CI 0.4–1.0, *p* = 0.06), whereas hysterectomy, lymphadenectomy, and adjuvant chemotherapy were not significantly associated with OS in the univariate model.

**Table 7:** Results of univariate and multivariate analyses for overall survival of MBOT patients (Cox proportional hazards model)

Univariate	Overall survival		
	Hazard ratio	95%CI	*p* value
Age (< 50 years vs. >= 50 years)	2.2	1.6–3.1	< 0.0001
Stage (IA,IB vs. IC)	2.7	2.0–3.7	< 0.0001
Hysterectomy	0.9	0.7–1.2	0.5
Omentectomy	0.6	0.4–1.0	0.06
Lymphadenectomy	0.9	0.5–1.6	0.7
Adjuvant chemotherapy (stage >= IC)	1.5	0.7–3.5	0.3

Multivariate	Overall survival		
	Hazard ratio	95%CI	*p*-value
Age (< 50 years vs. >= 50 years)	2.5	1.8–3.6	< 0.0001
Stage (IA,IB vs. IC)	2.7	1.9–3.6	< 0.0001
Hysterectomy	0.8	0.6–1.1	0.1
Omentectomy	0.6	0.4–1.0	0.05
Lymphadenectomy	0.8	0.4–1.5	0.5
Adjuvant chemotherapy (stage >= IC)	2.6	1.4–4.8	0.002

In the multivariate Cox regression analysis (Table 7), age ≥ 50 years and stage IC remained independent adverse prognostic factors (HR = 2.5, 95% CI 1.8–3.6, *p* < 0.0001; and HR = 2.7, 95% CI 1.9–3.6, *p* < 0.0001, respectively). Omentectomy was not independently associated with better overall survival (HR = 0.6, 95% CI 0.4–1.0, *p* = 0.05). Neither hysterectomy nor lymphadenectomy significantly affected survival. Interestingly, adjuvant chemotherapy for stage ≥ IC was associated with worse overall survival (HR = 2.6, 95% CI 1.4–4.8, *p* = 0.002**) after adjustment for confounders.

## Discussion

This study represents the largest nationwide, registry-based cohort analysis to date investigating mucinous borderline ovarian tumors (MBOTs). Using data from the JSOG Gynecologic Tumor Registry, it provides the most comprehensive overview of MBOTs in Japan. Between 2004 and 2018, a total of 12,766 patients were registered, demonstrating that MBOTs constituted 13–15% of all ovarian borderline tumors, with a remarkably stable incidence over time (Table 1). More than 90% of cases were diagnosed at FIGO stage I, and the median age was approximately 52 years—slightly older than that reported in European cohorts (40–45 years) [3, 4]. This finding suggests that MBOTs in Japan are more frequently detected in peri- or postmenopausal women, possibly reflecting differences in screening practices and surgical decision-making.

### Surgical practice patterns

Approximately half of all patients underwent hysterectomy, and over 60% underwent bilateral oophorectomy, whereas omentectomy was documented in 58% of cases after 2015 (Table 4). Lymphadenectomy was rare (< 10%) (Table 5), and adjuvant chemotherapy was used in only 2–3% of patients (Table 6). These data indicate that Japanese clinical practice is characterized by relatively conservative adjuvant treatment but still frequent use of hysterectomy, even in early-stage disease.

In contrast, Western guidelines and registry data—such as the cohort study of the AGO Study Group [3]—support fertility-sparing surgery for reproductive-age women without compromising survival [5, 6]. Our findings reinforce that radical surgery confers no survival advantage in patients with MBOTs.

### Comparison with previous multicenter studies

The present results closely align with those of a multicenter study by Gungorduk et al. [4], which analyzed 364 MBOT patients across 14 institutions in Turkey and Germany and found no independent prognostic effect of omentectomy, appendectomy, lymphadenectomy, or radical surgery on either progression-free or overall survival. In their study, the median age was 43 years, more than 75% of patients had stage IA disease, and the 5-year overall survival rate exceeded 95%.

Similarly, in our multivariate analysis, only older age (≥ 50 years) and advanced stage (IC vs. IA/IB) were identified as independent adverse prognostic factors (HR 2.5 and 2.7, respectively), whereas hysterectomy, lymphadenectomy, and chemotherapy provided no survival benefit (Table 7). It is also conceivable that the biological background of mucinous borderline ovarian tumors differs between younger and postmenopausal patients, including differences in driver genetic alterations. Mucinous tumors are frequently characterized by KRAS mutations; however, age-related differences in genomic complexity, hormonal milieu, immune surveillance, and accumulation of somatic alterations may influence tumor behavior and clinical outcomes. Such biological heterogeneity may partly explain the inconsistent prognostic impact of age reported in previous studies, including reports suggesting younger age as a risk factor for recurrence [7]. In this context, younger age may be more closely associated with recurrence risk, whereas older age may adversely affect overall survival through host-related factors rather than intrinsic tumor aggressiveness. Omentectomy showed a marginal association with improved survival (*p* = 0.05), which likely reflects selection bias or more complete staging rather than a therapeutic effect.

### Omentectomy and appendectomy

The role of omentectomy in MBOT remains controversial. In the study by Gungorduk et al., omental involvement was identified in only 1.4% of cases, and appendiceal involvement in 9.1%, mostly in macroscopically abnormal appendices [4]. Similarly, two other systematic reviews concluded that routine appendectomy is unnecessary unless gross abnormalities are observed [8, 9].

Although our registry did not capture appendectomy data, the low incidence of advanced disease and the excellent survival outcomes suggest that routine appendectomy and systematic omentectomy are unlikely to improve prognosis.

### Lymphadenectomy and adjuvant chemotherapy

Lymphadenectomy was performed in fewer than 10% of patients, consistent with its limited clinical utility given the extremely low incidence (< 2%) of nodal metastasis reported in prior studies [10, 11]. Our findings confirm that lymphadenectomy had no significant influence on overall survival (HR 0.8, *p* = 0.5).

Adjuvant chemotherapy was rarely administered and was associated with worse survival in the multivariate analysis (HR 2.6, *p* = 0.002), likely due to confounding by indication.

Previous meta-analyses have consistently shown no benefit from platinum-based chemotherapy for borderline ovarian tumors [12–14].

### Clinical implications and future directions

Taken together, our results support that the extent of surgical staging does not influence outcomes in MBOT patients. The excellent prognosis (> 98% 5-year survival) and very low incidence of extraovarian spread underscore the indolent biological nature of these tumors.

The main limitation of this study is its registry-based design, which relies on voluntarily reported data and thus contains missing information on some clinicopathological variables and outcomes. Detailed information regarding recurrence, including the timing of recurrence and specific recurrence patterns, is not available in the registry database. In addition, performance status was not collected. Therefore, while the present analysis allows evaluation of treatment selection trends according to patient age and treatment era, it is difficult to precisely investigate the exact causes of recurrence or death among patients with poor prognosis. Although the data accuracy cannot be fully guaranteed, the large sample size of over 12,000 cases provides sufficient statistical power and reliability.

In conclusion, fertility-sparing surgery should be recommended for younger women, and routine omentectomy, appendectomy, and lymphadenectomy should be reevaluated. Future registry-linked analyses integrating pathological review, molecular profiling, and clinical outcomes are warranted to refine surgical strategies for MBOTs and to clarify the prognostic relevance of histological subtypes (intestinal vs. seromucinous).

## Data Availability

Part of the data used in this study has been published annually in the Journal of Obstetrics and Gynaecology Research (JOGR) as part of the annual reports of the Japan Society of Obstetrics and Gynecology (JSOG).
